# The Experimental Study of the Validity of the Lagrange–Helmholtz Relationship in Geomagnetic Fields

**DOI:** 10.3390/s25051374

**Published:** 2025-02-24

**Authors:** Jing-jin Zhang, Yu-wei Xu, Zeng-zhou Yi, Qin-lao Yang, Jun-kun Huang, Fang-ke Zong

**Affiliations:** 1 College of Physics and Optoelectronic Engineering, Shenzhen University, Shenzhen 518060, China; zhangjingjin@szu.edu.cn (J.-j.Z.); 2200453034@email.szu.edu.cn (Y.-w.X.); 2300453058@email.szu.edu.cn (Z.-z.Y.); qlyang@szu.edu.cn (Q.-l.Y.); 2 North Night Vision Technology Co., Ltd., Kunming 650200, China; hjk@nvt.com.cn; 3 Institute of Advanced Science Facilities, Shenzhen 518106, China

**Keywords:** streak tube, streak camera, geomagnetic field, electronic optical imaging system

## Abstract

Streak cameras, known for their ultra-high spatiotemporal resolution, rely heavily on the spatial resolution capabilities of their core component, the streak tube, to ensure engineering stability. However, factors such as assembly inaccuracies and external magnetic fields, including geomagnetic interference, often cause deformation and shifts in the imaging plane. To enhance equipment stability and accelerate engineering advancements, a dual approach involving hardware improvements and computational imaging-based software corrections is essential. Future image reconstruction efforts in software require robust benchmarks; however, existing benchmarks are predominantly validated under idealized conditions, neglecting real-world interference factors. This study, grounded in electron optical imaging principles, experimentally confirms that the Lagrange–Helmholtz relationship remains valid within streak tube systems under geomagnetic field influences. These findings affirm that the imaging plane retains spatial resolution consistency despite such environmental disturbances. Consequently, the need for specific image orientations during reconstruction can be eliminated, enabling the development of more robust and efficient image reconstruction algorithms.

## 1. Introduction

The streak camera is an ultrafast imaging device renowned for its high spatial resolution and versatility, finding applications across diverse fields. These include laser scanning radars [1,2,3] utilizing streak tubes, studies on plant photosynthesis [4], the fluorescence lifetime decay analysis of biological samples [5,6,7,8,9], and investigations into superluminal propagation in matter through the integration of digital micromirror arrays and advanced image reconstruction methods [10].

In imaging systems, it is well established that the image plane typically exhibits curvature due to aberrations such as field curvature, and this phenomenon similarly affects streak cameras. The electro-optical conversion screen located at the tail end of the streak tube is commonly designed as either a flat phosphor screen or a spherical phosphor screen. However, the actual image plane of the electron beam deviates from a perfectly spherical surface. Consequently, even when using a spherical phosphor screen, complete alignment between the image plane and the screen is unattainable. This misalignment leads to variations in the projection of the electron beam: spots at nontangential positions on the phosphor screen appear larger than those at tangential positions. As a result, the final image exhibits a nonplanar resolution distribution across the entire field.

The geomagnetic field introduces additional complexity to the operation of streak tubes by causing shifts in the electron beam trajectory. Specifically, the focusing force of the electron beam within the focusing region is proportional to the square of its off-axis height. As a result, the geomagnetic field inevitably alters the focusing dynamics of the electron beam, leading to the deformation and displacement of the image plane [11].

Nevertheless, the Lagrange–Helmholtz relationship, which is universally valid in electron optical imaging systems, suggests that resolution uniformity should be achievable on the Petzval image plane. Based on this principle, it is hypothesized that the electron optical imaging system can maintain spatial resolution consistency despite geomagnetic interference. To validate this hypothesis, numerical methods were employed to calculate and track the electron trajectories inside the streak tube. These theoretical findings were further corroborated through experimental verification [12].

However, the experimental testing conducted in this work did not account for the influence of the geomagnetic field. If it can be demonstrated that the Lagrange–Helmholtz relationship remains valid under geomagnetic field interference, subsequent image reconstruction processes would no longer require the selection of specific images (e.g., those with particular streak tube orientations) for processing. This advancement would significantly enhance the robustness and efficiency of image reconstruction algorithms. This article aims to address this critical aspect of the study.

## 2. Theoretical Basis

The commonly accepted Lagrange–Helmholtz relationship in electronic optical imaging systems is shown in Equation (1) [13].

(1) M∗β=θ∗εo∗cos2θφtotal+εo∗cos2θ

**Figure 1:**
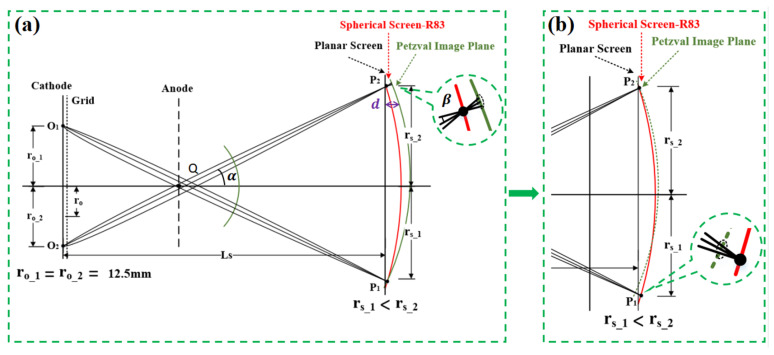
Test schematic: (**a**) P1 point focuses best; (**b**) P2 point focuses best.

In the formula, M represents the transverse magnification,$\varphi_{total}$ is the total anode voltage of the streak tube, $\varepsilon_{o}$ is the most probable energy of the photocathode material, and β is the image angle of an electron beam, as shown in Figure 1.

For the same photocathode material, its initial energy distribution adheres to either a Maxwell distribution or a Beta distribution. Additionally, the electron beam emission angles from each point on the cathode surface follow the Lambertian distribution, which means that the possibility of emission at any position on the surface is equal. Therefore, when the emission angle and energy are the same, the right-hand side of Equation (1) remains unchanged.

Under the influence of the Lorentz force exerted by the geomagnetic field, the landing point of the electron beam on the image plane inevitably deviates. This results in a slight change in the corresponding transverse magnification M.  This also also has a slight change ,∆M. Similarly, the aperture angle β of the electron beam at the image point undergoes a slight change, ∆β. Throughout this process, the aperture angle θ of the electron beam at the object point, the total anode acceleration voltage $\varphi_{total}$, and the axial energy $\varepsilon_{zo}$ of the object point do not change due to the presence of a magnetic field. Therefore, Equation (1) can be transformed into Equation (2).

(2) M+∆M∗β+∆βθ=εo∗cos2θφtotal+εo∗cos2θ

From Equation (2), it can be concluded that

(3) M∗Γ+∆M∗Γ+M∗∆Γ+∆M∗∆Γ=εo∗cos2θφtotal+εo∗cos2θ

By comparing Equations (1) and (3), it can be seen that, under the influence of the geomagnetic field, the normal condition for proving the validity of the Lagrange–Helmholtz relationship from a positive perspective is that it should satisfy

(4) ∆M∗β+M∗∆β+∆M∗∆β=0

However, as an imaging device with a high spatiotemporal resolution, the streak tube typically utilizes a low-initial-energy dispersed photocathode and structural parameters designed for a high total anode voltage. Under these conditions, according to Equation (1), the maximum order of magnitude on the right-hand side of the equation can be approximated as $10^{-2}$ (taking $\varepsilon_{o}=1\;eV$ and $\varphi_{total}=10\;kV$). In addition, considering the requirements for the detection area and the technological limitations of phosphor screens, the transverse magnification of the commonly used electron optical imaging system inside the streak tube is typically set to 1. Furthermore, the distortion is constrained so that it is less than 1%. Under these conditions, according to Equation (2), the minimum order of magnitude of β is also $10^{-2}$(rad). If Equation (1) still needs to hold under the influence of the geomagnetic field, ∆β should be 1%, which means that the measurement of the change in β should be $10^{-4}rad$. The measurement difficulty is extremely high.

For this purpose, this article employs a reverse proof approach, beginning with the assumption that Equation (4) holds under the influence of the geomagnetic field. Building on the author’s previous research, it has been established that the beam spot size remains consistent on the Petzval image plane. Using this foundation, the electron beam aperture angle β can be calculated by examining the relationship between the Petzval image plane and the beam spot on the planar phosphor screen [14]. Subsequently, it is possible to determine whether the product of β and the calculated transverse magnification M remain constant.

## 3. Testing Principles and Experimental Apparatus

As illustrated in Figure 1, the testing principle is based on the assumption that, under ideal conditions, the Petzval image plane of the streak tube should exhibit a parabolic spherical shape (the green curve in Figure 1, which is neither an ideal sphere nor a plane). However, under the influence of the geomagnetic field, the centre and shape of this image plane are subject to shifts and deformations. These distortions lead to asymmetry in the off-axis height, corresponding to the intersection points of the image and the phosphor screen, resulting in variations in both the magnification and the image-side aperture angle on opposite sides (as described in Equation (1)). Additionally, the geomagnetic field creates unequal axial positions for image points corresponding to the same object height, such as the edges on both sides of the cathode. Consequently, during the experimental process, the focusing voltage must be adjusted individually for these object points to achieve optimal focusing, as depicted in Figure 1.

In this article, two types of phosphor screen are utilized: a flat phosphor screen and a spherical phosphor screen with a curvature radius of 83 mm. The qualitative measurement method typically requires the use of reference materials. The purpose of employing two types of phosphor screen is to determine the parameters to be measured using the known shape function of the spherical phosphor screen. For instance, the image height and corresponding transverse magnification at any position on the spherical screen can be calculated from the centre of the image point based on the formula for a spherical cap [15]. Additionally, when the image point on the spherical phosphor screen is adjusted to its minimum size, the corresponding beam spot size is recorded after replacing the spherical screen with the flat phosphor screen under the same focusing voltage. Using these data, the image-side aperture angle β can be calculated from the relationship between the beam spots (as described in Equations (5)–(10)). In Figure 1, the red curve represents a spherical phosphor screen with a curvature radius of 83 mm, an effective diameter of 52 mm, and a visual height of 4 mm. The fibre optic panel used was processed to have a spherical input surface and a flat output surface. Subsequently, an S20 phosphor layer was prepared on the input surface using the centrifugal deposition method.

## 4. Experimental Testing and Analysis

**Figure 2:**
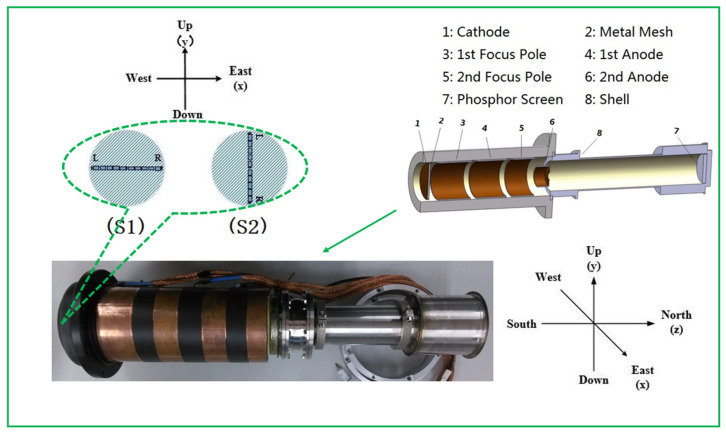
Streak tube for testing and testing plan: (**S1**) north–south orientation—horizontal placement; (**S2**) north–south orientation—turn right 90 degrees.

Due to the absence of a geomagnetic shielding darkroom in the laboratory, the relative tests in this article were conducted without geomagnetic shielding. The testing procedure is illustrated in Figure 2. Initially, the streak tube is oriented along the north–south axis, with the slit placed horizontally. After completing the first set of experiments, the streak tube is rotated 90 degrees clockwise around its axis, maintaining its north–south orientation but altering the slit orientation to be vertical.

Since the CCD image readout method operates line by line from top to bottom, the CCD is also rotated in the same direction as the streak tube to ensure that the readout direction remains consistent throughout the experiment.

**Figure 3:**
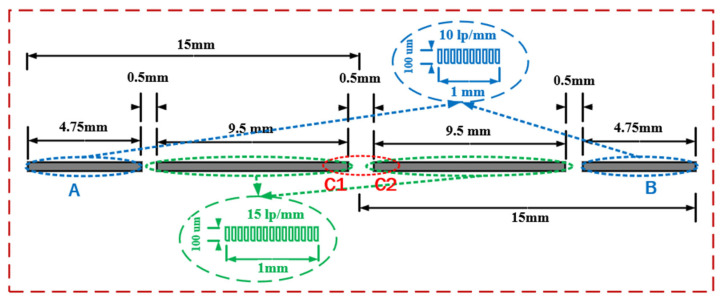
A schematic diagram of the reticle.

The streak tube used in this article is a si-electrode five-lane structure, with each electrode acting as shown in Figure 2. The entire tube is 424 mm long, with a transverse magnification of M=−1.35. The total anode acceleration voltage is 12 kV, and the effective diameter of the photocathode is 30 mm. The pattern of the reticle used for testing is shown in Figure 3, with an overall length of 30 mm, and divided into 4 modules. The central part has a resolution of 15 lp/mm, and the left and right sides have resolutions of 10 lp/mm. During the experimental testing, data were collected near the best rea on the spherical screen in the designated A and B regions on both sides. The C1 and C2 sections were selected to measure the magnification of the streak tube.

### 4.1. Measurement of Transverse Magnification

According to the definition of spatial resolution, a resolution of 10 lp/mm (or 15 lp/mm) on the object plane implies that the distance between the first peak and the tenth (or fifteenth) valley is 1 mm (as shown in Figure 3). On the image plane, this distance corresponds to the original size, multiplied by the transverse magnification.

The CCD used in this study is a scientific-grade CCD from the PI Corporation, USA [16]. This CCD has a resolution of 2048 × 2048, the area is 27 mm × 27 mm, and the corresponding size of a single pixel is 13.5 um. The image size on the image plane can be calculated by multiplying the difference in the number of pixels between the first peak and the tenth valley by the pixel size. The corresponding transverse magnification is then determined by comparing the calculated image size with the known object size.

**Figure 4:**
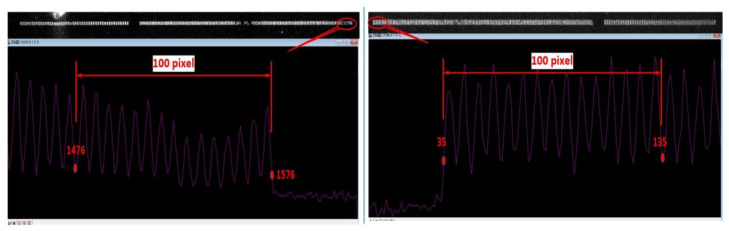
Image of scheme S1 on R83 mm spherical screen in Figure 3.

**Figure 5:**
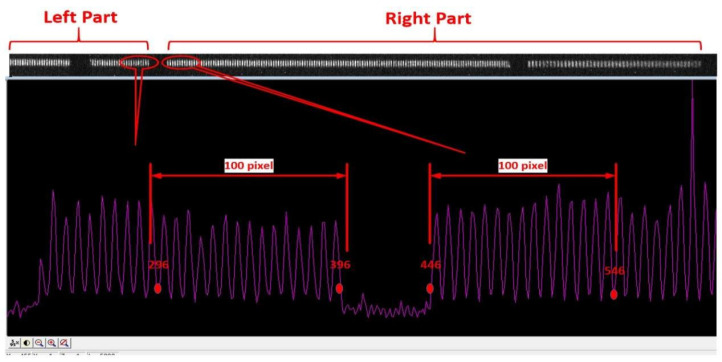
Image of scheme S2 on R83 mm spherical screen in Figure 3.

**Figure 6:**
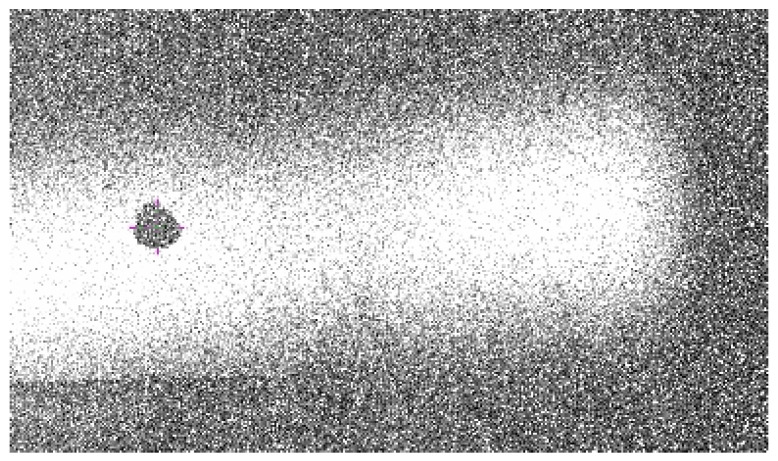
Centre marker point on the phosphor screen (black).

Figure 4 and Figure 5 display the images obtained from testing schemes S1 and S2, as described in Figure 2. The red numbers in the figures represent the pixel coordinates, with the origin as the reference point. The X-coordinate difference between the first peak and the fifteenth valley at the respective bases is measured as 101 or 100 pixels. By multiplying this difference by the pixel size of 13.5 um, the transverse magnification M≈1.35 can be calculated, which is consistent with the preset parameters. It should be noted that, due to the image size of the reticle (30 mm × 1.35 = 40.5 mm) exceeding the effective detection area of the CCD, in order to capture the full range of the image, the relative position between the CCD and the phosphor screen of the streak tube was adjusted during testing. Additionally, a marker was placed at the centre of the phosphor screen (opaque), and the electrode voltage ratio of the streak tube was adjusted to induce an abnormal focusing state of the internal electron beam. This procedure enabled the determination of the centre marker’s position on the phosphor screen, as shown in Figure 6. According to Figure 3, the left part of Figure 5 corresponds to the left side of the reticle pattern. However, a small interruption is observed in this area, caused by the obstruction from the centre marker on the phosphor screen.

### 4.2. Measurement of the Image-Side Electron Beam Aperture Angle

From the geometric relationships in Figure 1; the image-side electron beam aperture angle β can be calculated using Equations (4) and (5)

(5) $$R_{s}=R_{p}+\left(4-d\right)*\left(tg\left(\alpha +\beta \right)-tg\left(\alpha -\beta \right)\right)$$

In the formula, $R_{s}$ is the diameter of the beam spot on the planar phosphor screen, $R_{p}$ is the diameter of the beam spot on the planar phosphor screen, and d is the distance between the spherical screen and the planar screen, as shown in Figure 1a, for visual height. α is the elevation angle between the intersection point Q of the electron beam (emitted from the image height) and the corresponding object height on the phosphor screen relative to the axis. Meanwhile, β is half of the aperture angle of the spot on the phosphor screen relative to point Q, as depicted in Figure 1.

Expanding the trigonometric operation in Equation (5) yields

(6) Rp=Rs+2∗4−d∗tgβ∗1+tg2α1−tg2α∗tg2β

From Equation (6), we can obtain

(7) $$\beta =\left\{\begin{array}{c} \mathrm{atg}(\frac{\sqrt{4{K_{1}}^{2}{\mathrm{tg}}^{2}\alpha +{K_{2}}^{2}}-K_{2}}{{2*K}_{1}*{\mathrm{tg}}^{2}\alpha }),\;while\;tg\alpha \neq 0\\ \frac{K_{1}}{{2*K}_{2}},\;while\;tg\alpha =0 \end{array}\right.$$

where

(8) $$\left\{\begin{array}{c} K_{1}=R_{p}-R_{s}\\ K_{2}=2*\left(4-d\right)*\left(1+{tg}^{2}\alpha \right) \end{array}\right.$$

From Figure 1, it can be seen that

(9) tgα=(r0+rs)Ls+4−d

The formula for the spherical cap is

(10) $$d=83-\sqrt{83^{2}-{\left(r_{s}\right)}^{2}}\;(mm)$$

Using the above formula, the size of the electron beam aperture angle is first determined. Data are then collected from the C1 and C2 regions in Figure 4 to measure the intensity contrast. Next, the modulation transfer function is applied, using the simplified Formula (11), to calculate the corresponding beam spot diameter R (i.e., $R_{p}$ or $R_{s}$) and then process it according to Equations (7)–(10) [13,17]. The original experimental data (intensity) of the schemes are shown in Table 1 and Table 2, and results calculated according to the above equations of each scheme are shown in Table 3 and Table 4:

(11) R=−logIMax−IMinIMax+IMin−2∗Inosπ∗f

In the formula, $I_{Max}$, $I_{Min},$ and $I_{nos}$ represent the peak, valley, and background noise of the streak pattern in the experimental image, respectively, while f represents the spatial resolution corresponding to the reticle pattern in Figure 3.

From Table 3 and Table 4, it is evident that, in both schemes one and two, the transverse magnification M of the streak tube remains unchanged, while the image-side electron beam aperture angle varies. However, the product of the parameters obtained in the two schemes does not conform to the relationship described by Equation (1). Our analysis suggests that this discrepancy arises because the Petzval image plane of the streak tube is not an ideal spherical surface but rather a parabolic-shaped surface. In an ideal scenario, the distance between points on either side of the image plane centre and the centre itself is linear. However, due to the influence of the geomagnetic field, the overall position of the electron beam is shifted and deformed, further intensifying the nonlinearity in these distances. Consequently, if the ratio between image height and object height is used to calculate the transverse magnification, the transverse magnification at each point will inherently exhibit nonlinearity. Similarly, the calculated image-side aperture angle of the electron beam will also be nonlinear. From Table 1 and Table 2, it can be observed that, in various schemes shown in Figure 2, the image-side aperture angle of the electron beam remains nearly constant. This consistency indicates that the Lagrange–Helmholtz relationship holds even under the influence of the geomagnetic field.

## 5. Conclusions

In addition to the influence of assembly process accuracy, the streak tube, as an imaging system, is inevitably affected by optical aberrations. Furthermore, as its primary target is the electron beam, it is subject to interference from the surrounding magnetic field. These factors result in the deformation and displacement of the image plane, leading to spatial resolution inconsistencies across the image plane and limiting the hardware’s working stability. To enhance the engineering stability of the streak camera in the future, it will be essential to adopt computational imaging techniques such as image reconstruction. These methods can mitigate the effects of hardware-induced limitations and improve the overall performance and reliability of the system.

The purpose of computational reconstruction is to achieve a flat-field resolution across the image. In previous research, the author proposed that one of the criteria for evaluating reconstruction results is the imaging consistency on the Petzval image plane of the electron optical imaging system within the streak tube. However, this criterion was established without considering the influence of the surrounding environment.

This article experimentally demonstrates that the Lagrange–Helmholtz relationship in electron optical imaging systems remains valid under the influence of the geomagnetic field. This finding confirms that the resolution on the imaging plane of the streak tube maintains consistency. Consequently, in subsequent image reconstruction processes, it is not necessary to select specific images (e.g., those with specific streak tube orientations) for processing. This improvement makes the image reconstruction algorithm more robust and efficient.

The limitation of this article is that we cannot directly prove the validity of the LG formula in a positive direction. As mentioned earlier, due to the limitations of the pixel size of the image acquisition device and the geomagnetic shielding cavity, the error is relatively large. At the same time, it also indicates that if we want to further improve the accuracy of the results, we should adopt a CCD acquisition system with a higher resolution and install a geomagnetic shielding cavity. This work also needs to carry out in the future.

## Figures and Tables

**Table 1:** The original experiments data (intensity) of Scheme S1.

Collection Frequency		C1			C2			P1			P2		
		Max	Min	Noise	Max	Min	Noise	Max	Min	Noise	Max	Min	Noise
Spherical Screen (R83)	1	4737	2112	1448	4011	1548	1107	5814	1928	1623	4302	1859	1753
	2	4382	2112	1442	3576	1548	948	5702	1928	1633	4459	2002	1677
	3	3547	1746	1449	3759	1557	994	6118	2131	1666	4733	1961	1662
	1	8583	6767	5647	5153	3758	1746	9602	6219	5341	6414	3554	2739
Planar screen	2	8745	6948	5470	5073	3758	1724	9248	6277	5296	6587	3554	2803
	3	9085	6948	5610	5013	3510	1788	9842	6277	5388	6579	3550	2792

**Table 2:** The original experiments data (intensity) of Scheme S2.

Collection Frequency		C1			C2			P1			P2		
		Max	Min	Noise	Max	Min	Noise	Max	Min	Noise	Max	Min	Noise
Spherical screen (R83)	1	6016	2877	2226	6211	3026	2226	6920	2889	2517	5389	3074	2856
	2	5833	2877	2240	6493	2944	2240	6831	2889	2510	6337	3074	2826
	3	6308	2955	2241	6349	2944	2241	6800	2892	2508	6198	3174	2861
	1	6369	4079	3145	6743	4500	3145	7145	3256	2518	7811	4732	4225
Planar screen	2	5955	4470	3089	6766	4500	3089	7341	3287	2578	7938	4732	4243
	3	6182	4122	2994	6283	4294	2994	7117	3287	2494	7934	4672	4172

**Table 3:** Scheme S1.

	$R_{p}$	$R_{s}$	$\mathrm{Factor}\;K_{1}$	Object Height	Image Height	M	d	$\mathrm{Factor}\;K_{2}$	β
C1	0.0490	0.026	0.023	0.25	−0.378	−1.35	0.0008608	7.9983	0.002876
C2	0.0486		0.0226	−0.25	0.918		0.005077	7.9899	0.002829
P1	0.0423		0.0163	10.25	−13.959		1.1822	5.6537	0.002889
P2	0.0412		0.0152	−10.25	14.769		1.3246	5.3693	0.002837

**Table 4:** Scheme S2.

	$R_{p}$	$R_{s}$	$\mathrm{Factor}\;K_{1}$	Object Height	Image Height	M	d	$\mathrm{Factor}\;K_{2}$	β
C1	0.0375	0.026	0.0115	0.25	2.6055	−1.35	0.04091	7.9185	0.001446
C2	0.0382		0.0122	−0.25	3.3615		0.06810	7.8644	0.001554
P1	0.0362		0.0102	10.25	−10.328		0.6450	6.7255	0.001517
P2	0.0332		0.0072	−10.25	16.942		1.7476	4.5231	0.001592

## Data Availability

If necessary, the author of this article may provide raw data to readers as appropriate.
